# Endogenous Bacteremia Caused by Intestinal Colonization of Carbapenem-Resistant *Enterobacteriaceae* (CR*E*) in Immunocompromised Children

**DOI:** 10.3390/tropicalmed8080402

**Published:** 2023-08-07

**Authors:** Nasim Almasian Tehrani, Leila Azimi, Shahnaz Armin, Neda Soleimani, Fatemeh Fallah, Abdollah Karimi, Bibi Shahin Shamsian, Shiva Nazari, Masoud Alebouyeh

**Affiliations:** 1 Pediatric Infections Research Center, Research Institute for Children’s Health, Shahid Beheshti University of Medical Sciences, Tehran 1546815514, Iran; n.a.tehrani@ut.ac.ir (N.A.T.); arminsh_2000@yahoo.com (S.A.); dr_fallah@yahoo.com (F.F.); dr.y.karimi@gmail.com (A.K.); shnazari2000@yahoo.com (S.N.); 2 Department of Microbiology and Microbial Biotechnology, Shahid Beheshti University, Tehran 1983969411, Iran; n_soleimani@sbu.ac.ir; 3 Pediatric Congenital Hematologic Disorders Research Center, Research Institute for Children’s Health, Shahid Beheshti University of Medical Sciences, Tehran 1546815514, Iran; shahinshamsian@gmai.com

**Keywords:** antimicrobial resistance, endogenous infection, carbapenem-resistant Enterobacteriaceae, children, bacteremia

## Abstract

Objective: Carbapenem-resistant Enterobacteriaceae (CR*E*) infection is life-threatening, especially for immunocompromised children. The source tracking of CR*E* could prevent bacteremia during hospitalization. In this study, the intestinal colonization of CR*E* and their translocation to blood were investigated. Methods: Stool samples from immunocompromised pediatric patients were collected after admission, and secondary stool and blood samples were collected in case of fever. After CR*E* phonotypic detection, the OXA-48, NDM-1, VIM, IMP, and KPC genes were detected by PCR. Enterobacterial Repetitive Intergenic Consensus Polymerase Chain Reaction (ERIC-PCR) was used to determine the phylogenic relatedness of the blood and fecal isolates. Results: Bacteremia was recorded in 71.4% of the patients. Enterobacteriaceae spp. were recorded in 100% of the stool samples and 31% of the blood samples. The correlation between the length of stay (LOS), days of fever, chemotherapy regimens, and death rate was significant (*p*-value ≤ 0.05). OXA-48 was present in all CR*E* isolates in both the primary and the secondary stool samples and the blood samples. According to the phylogenetic data, 58.33% of the patients with bacteremia had identical blood and stool isolates. The death rate was 24.4% in children with CR*E* bacteremia. Conclusions: The primary intestinal colonization with CR*E* in immunocompromised pediatrics and their translocation to blood was established in this study. The implementation of infection control programs and the application of infection prevention strategies for immunocompromised children is necessary.

## 1. Introduction

The gastrointestinal tract (GIT) of humans is considered the main source of enteric pathogens [1]. *Enterobacteriaceae* is a family of bacteria that belongs to Gammaproteobacteria, which are members of the gut microbiota. They can harbor different virulence genes that will convert them to life-threatening pathogens for humans [2,3]. These bacteria are known as the cause of bacteremia, diarrhea, surgical site infections, pneumonia, and urinary tract infections in both hospitalized and non-hospitalized people in all age groups [4,5]. Children, old patients, immunocompromised patients, and those with immune deficiency are more prone to infection with *Enterobacteriaceae*. Intrinsic and acquired resistance to different types of antimicrobials are reported in most genera of this family, which would suggest they are the main cause of treatment failure in clinical settings [6,7]. Conjugative plasmids, as the most common transgenic elements, are responsible for the transfer of several resistance genes determinants among members of *Enterobacteriaceae* that make them resistant to related antibiotics and limit treatment options upon disease onset [8].

Source tracking can be used as a tool for understanding the transmission route of pathogenic microbes among patients in a hospital. The tracking of clinically important pathogenic bacteria could enable the infection control team to prevent the spread of the responsible pathogen. Different strategies upon detection of the infection source, either endogenous or exogenous, could be designed depending on the types of the responsible organism, the manner of its spread, and resistance to antimicrobials [9,10]. Immunocompromised patients are predisposed to hospital-acquired infections (HAIs) owing to the decreased immune function induced by the medication. Patients undergoing chemotherapy can experience gastrointestinal mucositis that may facilitate the local translocation of the gut microbiota, mainly *Enterobacteriaceae*, to the blood, which will cause bacteremia or sepsis in severely ill patients [1,11]. An appropriate multidisciplinary approach for the management of adverse effects of medications and the reduction in mortality of immunocompromised or cancer patients seems necessary [12].

Bloodstream infections due to carbapenem-resistant *Enterobacteriaceae* (CR*E*) are associated with a high hospital mortality rate that is challenging to clinicians [13,14]. Although carbapenems, the strongest member of the beta-lactam antibiotics, have a wide range of activity against many Gram-negative bacteria [15], the production of carbapenemase is known as the main mechanism of resistance to these antibiotics. Out of four known classes of carbapenemases, members of class A, such as *KPC*, class B, such *NDM-1*, and class D, which are known as OXA-type, are more common causes of treatment failure in different countries [16,17]. Current data about the outcomes of CR*E* infection in children, mainly those with immunocompromised conditions, and sources of their transmission are scant. Frequent hospitalization is a risk factor for CR*E* infection in children with underlying diseases. These bacteria can be acquired during hospitalization or through the food chain, and the environmental sources and can persist for life in the gastrointestinal tract after colonization [18,19]. The previous history of carbapenem administration, both as prophylaxis or as therapeutic regimens, could enrich the colonized CR*E* leading to dysbiosis of the GIT in these patients [20]. Immunocompromised children who undergo chemotherapy and stem cell transplantation are more vulnerable to bloodstream infections due to long hospital stays, gastrointestinal tract mucosal barrier injury, and impaired innate immunity [21]. Although early prophylaxis treatment against bacteremia is clinically important for these children, there should be more consideration for the type of the prescribing antimicrobials [22]. The detection of sources of bloodstream infection in these patients can help clinicians to manage the infection and prevent its recurrence during different courses of hospitalization and therapies. Accordingly, the current study aimed to investigate the occurrence of bacteremia with CR*E* in immunocompromised children and analyze the impact of prophylactic and chemotherapeutic drugs, length of hospital stays, and intestinal colonization with CR*E* on the occurrence of this infection. Antimicrobial susceptibility testing and molecular typing methods were used to show the similarity of resistant and genetic patterns among the fecal and blood isolates in each patient and determine common sources of the infection among the patients at different wards.

## 2. Material and Methods

### 2.1. Patients and Sampling

Immunocompromised children who were admitted to the bone marrow transplantation (BMT) and oncology wards of Mofid Children’s Hospital, Tehran, Iran, were included in this cross-sectional study from August 2020 to August 2021. The types of cancer, underlying diseases, and medications for these patients were recorded based on the documented clinical diagnostics in their medical files. Sampling was performed during two main periods from children who did not receive antimicrobial drugs at least one week before admission. Stool samples were collected during the first 24 h of admission, before receiving antibiotic prophylaxis. If the patients had fever, a second stool sample and blood sample were ordered. The stool samples were immediately transferred to the microbiology laboratory in the Pediatric Infections Research Center for further processing and culture. The blood samples were directly inoculated into Pediatric BACTEC vials and sent to the microbiology laboratory, as described below. Demographic information of the patients was collected through a questionnaire and the parent or guardian of each child signed an informed consent form for this study.

### 2.2. Bacterial Culture and Characterization

To characterize members of *Enterobacteriaceae* in feces and blood samples, the stool samples were homogenized in normal saline and 100 µL of each suspension was cultured on blood agar (Merck, Darmstadt, Germany) and MacConkey agar (CONDA, Tudela, Spain) and incubated in the air at 37 °C for 24 h. Growth of the colonies was followed after 24 h and screened for the *Enterobacteriaceae* family. Conventional biochemical tests, such as fermentation of sugars, reduction of indole, motility, production of acetyl methyl carbinol using the Voges–Proskauer test, use of citrate as the sole carbon source, decarboxylation of amino acids, and urease activity, were performed based on standard protocols [23]. Microgen biochemical identification systems (Microgen, Camberley, UK) were used for the characterization of some of the isolates that the routine identification method did not identify. Blood culture was performed using the BACTEC system (Becton Dickinson Diagnostic Instrument Systems, Sparks, MD, USA). The inoculated pediatric BACTEC bottles were followed up in the device for 7 days, until a positive alarm was reported. The recorded positive samples were used for the isolation and characterization of responsible pathogens as described for the stool samples. After characterizing the *Enterobacteriaceae* strains, fresh cultures were used for conservation in a −70 °C freezer for further analysis. The antimicrobial susceptibility testing was performed for all the conserved *Enterobacteriaceae* isolates from the primary and secondary stool samples and blood samples.

### 2.3. Antimicrobial Susceptibility Testing

Susceptibility of the fecal and blood isolates in febrile children was determined against imipenem (10 µg), meropenem (10 µg), amikacin (30 µg), gentamycin (10 µg), ceftazidime (30 µg), levofloxacin (5 µg), cefepime (30 µg), cefotaxime (30 µg), aztreonam (30 µg), and tigecycline (15 µg) using a disk diffusion method according to the latest updates of Clinical and Laboratory Standard Institute (Performance standards for antimicrobial susceptibility testing. 31st ed. ClSI supplement M100. Clinical and Laboratory Standards Institute, Wayne, PA, USA, 2021). Members of *Enterobacteriaceae* that showed resistant phenotype to imipenem or meropenem were primarily considered as CR*E* and selected for further analysis. *E. coli* ATCC 25922 was used as a quality control strain in every test run.

### 2.4. Detection of Carbapenemase Genes in the CRE Isolates

DNA extraction based on the boiling method was performed on fresh subcultures of the blood and stool isolates with the CR*E* phenotype. Primers targeting *blaOXA-48*, *blaNDM-1*, *blaVIM*, *blaIMP*, and *blaKPC* genes were used for the screening of carbapenemases. *K. pneumoniae* strain OM479563 for *blaNDM-1* and laboratory-confirmed strains of *K. pneumoniaea* for the other carbapenemase genes (PIRC-K.P.90.KPC, PIRC-A.M.205.OXA-48, PIRC-A.M.209.VIM, PIRC-A.H.34.IMP) were used as positive controls in this study. Nucleotide sequences of the primers and PCR conditions of each gene are shown in Table 1.

### 2.5. Molecular Typing by ERIC-PCR

To determine the similarity and clonal relatedness of CR*E* isolates in the collected blood and stool samples of hospitalized children, ERIC-PCR was used as previously described [30,31]. The ERIC-PCR primer and amplification conditions are shown in Table 1. The phylogenetic relationship of CR*E* strains based on antimicrobial susceptibility patterns, carriage of carbapenemase genes, and ERIC-PCR patterns was determined using NTSyS software version 2.02. The strains with 100% homology were considered identical, while others with >80% and 70–80% homology were defined as related and similar strains, respectively [32].

### 2.6. Data Analysis

Statistical analysis was performed by SPSS software version 23. To determine the relationship between antimicrobial susceptibility and underlying disease, gender, age, history of previous hospitalization, length of stay, and death rate, Student’s *t*-test was used. A *p*-value ≤ 0.05 was considered statistically significant.

## 3. Results

### 3.1. Patients

Overall, 63 admitted immunocompromised children (12 from the Bone Marrow Transplantation ward and 51 from the Oncology wards) were included in the current study from August 2020 to August 2021. The median age of patients was 6.8 years old (range between 1 and 15 years of age). Boys and girls constituted 55.5% (35/63) and 44.4% (28/63) of the cases, respectively. Among the admitted children, fever was recorded in 54 patients after 48 h of hospital stay (54/63, 85.7%). The second stool samples and blood samples were collected from 45 of the febrile children according to medical orders. The presence of underlying diseases, types of medication, and demographic data of these children are shown in Section 3.4.

### 3.2. Bacterial Isolates

In this study, a total of 63 patients were included. Fever was recorded in 85.7% (54/63) of these patients. Blood culture was ordered for 45 of the febrile children. Among the 45 cultures of the primary and secondary stool samples and blood samples of these patients, members of *Enterobacteriaceae* were detected in 95.55% (43/45), 97.77% (44/45), and 31.11% (14/45) of samples, respectively. *E. coli* was the most common isolate in the primary and secondary stool samples, and *Klebsiella* spp. were the most frequent strains isolated from the blood samples. *Enterobacter agglomerans*, *Escherichia herminie*, *Enterobacter cloacae*, *Serratia rubidaea*, *Citrobacter sedlakii*, and *Citrobacter gillenii* were the other isolates characterized in the blood samples. The diversity and the frequency of the isolates of the studied samples are presented in Table 2. The same bacterial species was detected in 26.66% (12/45) of the blood and stool samples. Other microorganisms such as *Acinetobacter* spp. *Pseudomonas* spp. and staphylococci were also detected in the samples.

### 3.3. Antimicrobial Resistance and Frequency of CRE among the Blood and Stool Samples of Febrile Children

Antimicrobial resistance to 10 different antimicrobials was measured among members of *Enterobacteriaceae*. Generally, in the blood isolates, the highest resistance rate was detected against imipenem, meropenem, and cefotaxime (92.8%, 92.8%, 92.8%, respectively). The highest resistance rate of the strains in the primary and secondary stool samples was detected against cefotaxime (75% and 82.2%, respectively). Resistance to imipenem and/or meropenem, which was characterized as CR*E*, was detected in 92.8% of the blood samples, 31.83% of the primary samples, and 42.2% of the secondary stool samples (Table 3). A diagram of all the sampling stages is shown in Supplementary Figure S1.

### 3.4. Diversity of Carbapenem Resistance Genes among CRE

**Figure 1:**
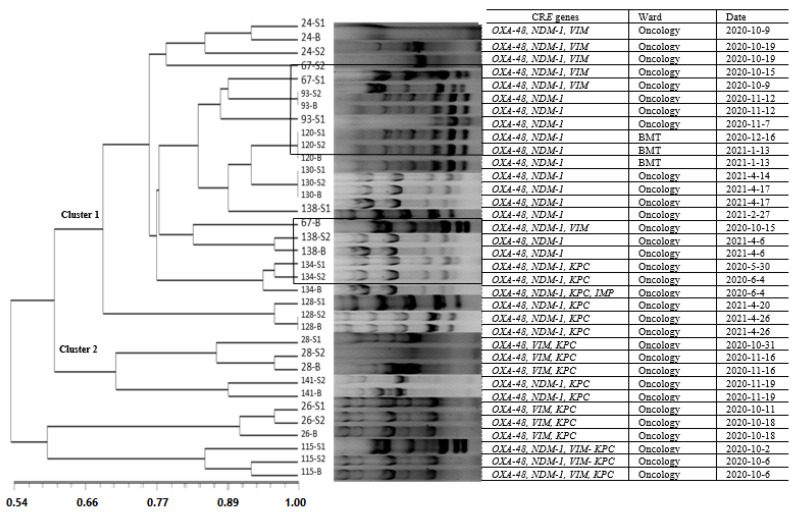
ERIC-PCR products and dendrogram of CR*E* strains isolated from primary and secondary fecal and blood samples of children in oncology and transplantation units. Legend. The scale bar represents the similarity coefficient and distance among the strains.

The carriage of carbapenemase-producing genes was screened among the strains of CR*E* in each patient. Accordingly, *OXA-48* (14/14, 100%; 18/19, 94.73%; and 13/13, 100%, respectively), *NDM-1* (10/14, 71.42%; 13/19, 68.42%; and 11/13, 84.61%, respectively), *VIM* (7/14, 50%; 8/19, 42.1%; and 7/13, 53.84%, respectively), *KPC* (4/14, 28.57%; 4/19, 21.05%; and 4/13, 30.76%, respectively), and *IMP* (2/14, 14.28%; 1/19, 0.05%; and 0, respectively), were detected in the primary stool, secondary stool, and blood samples, respectively. Co-carriage of carbapenemase genes was detected in CR*E* both in the feces and blood isolates Figure 1. *OXA-48/NDM-1* in *K. pneumonia* (9/24, 37.5%) and *OXA-48/NDM-1*/*VIM* in *E. coli* (6/21, 28.57%) were among the most frequent carbapenemase genes that coexisted in CR*E* isolates from the stool and blood samples. The association of CR*E* isolation and bacteremia with demographic data, medication, underlying diseases, LOS, and death is shown in Table 4.

### 3.5. Molecular Typing of CRE in the Blood Samples and Their Similarity with the Stool Isolates

As shown in Figure 1, among the patients from whom the same species of CR*E* were isolated from the blood and stool samples, molecular identity was detected in 33.33% (4/12) of them. Bacteremia with related or similar strains from the primary and second stool samples was found in 50% (6/12) of the patients, and bacteremia with similar strains or unrelated fecal strains from both the primary and secondary stool samples were detected in 8.33% (1/12) and 8.33% (1/12), respectively. No homology among the CR*E* isolates between the studied wards was shown in this study. More details are shown in Figure 1.

## 4. Discussion

One of the problems of CR*E* bacteremia is the unknown source of the infection, which can be either endogenous or exogenous. Immunocompromised pediatrics are very susceptible to endogenous infections due to the loss of their natural defense barrier [33]. In this study, we detected the source of endogenous bacteremia caused by CR*E* in immunocompromised children for the first time in the world. In addition, the correlation between gender, age, fever days, length of stay, underlying diseases, wards of the hospital, prophylaxis antibiotics, alternative antibiotics, chemotherapy drugs, death of patients, and CR*E* bacteremia was evaluated. Our findings showed significant correlations between length of hospital stay, fever days, death rate, and CR*E* bacteremia among the children. Consistent with our results, the correlation of mortality rate, length of hospital stay, and CR*E* bacteremia among children was shown in two studies in India and Spain [20,34]. Our results showed a high rate of mortality in children with CR*E* bacteremia that underwent chemotherapy or those who were prescribed immunosuppressive drugs, which was similarly presented in a study of seven Italian pediatric centers [9,35]. In our study, the most common species isolated from primary fecal samples was *E. coli* (63.63%); 28.57% of them were carbapenem-resistant. Based on a study of stool samples of children admitted to the outpatient clinic of a hospital in Shanghai in 2019, a carriage rate of 3.6% was shown for CR*E*. The most common CR*E* isolates belonged to *E. coli* (37.5%) and *K. pneumonia* (37.5%), while *E. cloacae* (18.8%), *Enterobacter aerogenes* (3.1%), and *Raoultella planticola* (3.1%) were also characterized [36]. In our study, *E. coli* was present at the highest frequency among strains isolated from secondary fecal samples during fever onset (62.22%), of which 35.71% of them were CR*E*. Despite numerous studies that have been performed on the intestinal colonization of CR*E* in patients with different clinical situations, according to our knowledge, there are no follow-up studies to show the risk of this colonization in bacteremia, especially among children during the hospitalization period. In the present study, members of *Enterobacteriaceae* constituted 31.11% of the blood isolates in febrile children, of which 100% of them were carbapenem-resistant. The most common *Enterobacteriaceae* strains belonged to *K. pneumoniae* (50%) and *E. coli* (21.42%) in the blood samples. Similar to our findings, in a study in India that was performed in *Enterobacteriaceae* in the blood samples of children, *K. pneumoniae* (76%) and *E. coli* (23%) were the most common strains, of which 97% of the strains were carbapenem-resistant [20]. It seems that CR*E* bacteremia is not only limited to immunocompromised children or those with cancer. In a study of adult cancer patients in Iran, 51% of *E. coli* and *K. pneumoniae* isolates in blood samples were resistant to carbapenems [37].

According to the antimicrobial susceptibility patterns of the strains isolated from fecal and blood samples of patients with bacteremia, the highest and the lowest resistance in primary fecal samples was observed against cefotaxime (75%) and tigecycline (6%), respectively. In the isolates from secondary stool samples, the highest and the lowest resistance phenotypes were against ceftazidime (86.6%) and tigecycline (15.5%), respectively. While resistance to third-generation cephalosporins was common in the strains isolated from stool samples, the highest resistance patterns of the blood samples were observed against carbapenems (28.88%) and cefotaxime (28.88%). Similarly, tigecycline showed the lowest resistance among the blood isolates (6.66%). The high rate of intestinal colonization with CR*E* in pediatric patients could be explained through their history of multiple admissions in different parts of hospitals, including surgery, respiratory, Pediatric Intensive Care Unit (PICU), and infectious units, plus a history of a variety of broad-spectrum antimicrobial drug usage, including carbapenems. In support of this finding, the results of a study on critically ill adult patients showed that CR*E* colonization at the time of ICU admission, previous hospitalization, or ICU admission are predictors of 90-day mortality [38].

The most frequent carbapenemase was *OXA-48* (100%), which was similar to previous reports from the Middle East and Iran. In a study on immunocompromised patients conducted in Iran, 96% of CR*E* carried *OXA-48* [39]. In another study in Egypt, 58% of *Enterobacteriaceae* strains isolated from immunocompromised children with cancer carried the *OXA-48* gene [40]. The high percentage of *OXA-48* in clinical isolates of *Enterobacteriaceae* indicates its widespread distribution, which is important epidemiologically. The second most common carbapenemase gene in the present study was *NDM-1* (74.81%). This gene is located on a plasmid that can harbor resistance genes to other antibiotics. *NDM-1* plays an important role in HAI, so it is known as one of the most important carbapenemase enzymes that can endanger public health [41,42,43]. While the presence of two carbapenemase genes was reported among *Enterobacteriaceae*, the co-carriage of three carbapenemase genes in different combinations among different genera, including *OXA-48*, *VIM,* and *KPC* in *K. oxytoca*, *OXA-48*, *VIM*, *KPC* and *NDM-1* in *K. pneumonia*, *OXA-48*, *VIM* and *NDM-1* in *K. pneumonia* and *OXA-48*, *VIM,* and *NDM-1* in *E. coli*, was reported for the first time.

Based on the molecular epidemiology results, identical patterns with 100% homology between the blood and the stool samples were detected among 33.33% of the patients. Changes in the CR*E* molecular types occurred in a few patients during the two sampling periods, which highlighted the importance of intestinal colonization at the time of admission and its risk for bacteremia and mortality. The presence of common source of infection with CR*E* was shown in this study through observed similarity in the ERIC-PCR molecular patterns of the bacterial isolates in fecal and blood samples between two patients at oncology and BMT wards. The patients were hospitalized for similar periods with similar genotypes and antibiotic resistance patterns. Our results also showed similar ERIC-PCR patterns between two patients in the oncology ward that were hospitalized in a similar time period; however, the isolates did not have identical CR*E* genes.

There are pros and cons about the current study. According to our knowledge, this is the first study in immunocompromised children that has investigated the primary intestinal colonization of CR*E* and its impact on bacteremia during the hospitalization. Moreover, the association of clinical data, such as antibiotic regimens, treatment periods, and underlying diseases, with the microbiological findings were analyzed in this study, which highlighted the link between CR*E* bacteremia and chemotherapy, the length of hospital stays, and death in this population. However, the lack of some information, such as the history of previous hospitalization and infection with CR*E*, and inability to perform the comparison of patients in the Oncology/chemotherapy and Transplantation wards with those referred to the other wards were among main limitations at the time of this study. The follow-up of the patients and study of the situation of carrier status of CR*E* could provide valuable data about the impact of hospital stays, and antibiotics and chemotherapeutic regimens for immunocompromised children. The application of high-throughput techniques for molecular typing is recommended instead of ERIC-PCR, which was not possible regarding to the time and budget constraints. Further studies are needed to detect and control sources of CR*E* in the community and in hospital settings and control its spread.

## 5. Conclusions

Our findings showed the primary intestinal colonization with CR*E* in immunocompromised pediatrics and their translocation to blood, which is a risk factor for mortality. The co-carriage of several carbapenem resistance genes in some strains, such as *K. oxytoca*, *K. pneumoniae*, and *E. coli*, indicates the role of mobile genetic elements in the emergence of new life-threatening CR*E* variants. A history of multiple hospitalization and the length of hospital stay was correlated with CR*E* colonization and bacteremia in these children, which show the necessity of designing proper prophylactic regimens in oncology and chemotherapy wards.

## Figures and Tables

**Table 1:** Nucleotide sequences of the primers used for detection of carbapenemase genes and ERIC-PCR typing.

Carbapenemase Genes	PCR Product (bp)	Sequence (5′–3′)	PCR Conditions	Reference
OXA-48	392	F: CCAAGCATTTTTACCCGCATCKACC R: GYTTGACCATACGCTGRCTGCG	Initial denaturation at 95 °C for 1 min; 30 cycles of denaturation at 95 °C for 30 s, annealing at 55 °C for 30 s, extension at 75 °C for 1 min; and final extension at 72 °C for 7 min	[24]
NDM-1	129	F: CCCCGCCACACCAGTGACANCTC R: GTAGTGCTCAGTGTGGGCAT	Initial denaturation at 95 °C for 1 min; 32 cycles of denaturation at 95 °C for 30 s, annealing at 61 °C for 30 s, extension at 7 °C for 1 min; and final extension at 72 °C for 5 min	[25]
VIM	390	F: GATGGTGTTTGGTCGCATA R: CGAATGCGCAGCACCAG	Initial denaturation at 94 °C for 10 min; 35 cycles of denaturation at 94 °C for 30 s, annealing at 61 °C for 40 s, extension at 72 °C for 1 min; and final extension at 72 °C for 7 min	[26]
IMP	139	F: TTGACACTCCATTTACDG R: GATYGAGAATTAAGCCACYCT	Initial denaturation at 94 °C for 10 min; 33 cycles of denaturation at 94 °C for 30 s, annealing at 61 °C for 40 s, axtension at 72 °C for 40 s; and final extension at 72 °C for 7 min	[27]
KPC	636	F: CTGTCTTGTCTCTCATGGCC R: CCTCGCTGTGCTTGTCATCC	Initial denaturation at 94 °C for 5 min; 32 cycles of denaturation at 94 °C for 35 s, annealing at 62 °C for 35 s, extension at 72 °C for 32 s; and final extension at 72 °C for 5 min	[28]
ERIC-PCR		F: ATGTAAGCTCCTGGGGATTCAC R: AAGTAAGTGACTGGGGTGAGCG	Initial denaturation at 94 °C for 3 min; 35 cycles of denaturation at 94 °C for 1 min, annealing at 48 °C for 1 min, extension at 72 °C for 2 min; and final extension at 72 °C for 5 min	[29]

**Table 2:** Diversity and frequency of members of *Enterobacteriaceae* in stool and blood samples from febrile immunocompromised children.

Bacterial Species	Primary Stool ^a^ N = 44 (n, %)	Secondary Stool ^b^ N = 45 (n, %)	Blood ^c^ N = 45 (n, %)
*Escherichia coli*	28, (63.63)	28, (62.22)	3, (6.66)
*Klebsiella pneumoniae*	9, (20.45)	12, (26.66)	7, (15.55)
*Klebsiella ozaenae*	1, (2.27)	0, (0)	-
*Enterobacter. agglomerans*	1, (2.27)	0, (0)	-
*Escherichia herminie*	1, (2.27)	1, 2.22)	-
*Klebsiella oxytoca*	1, (2.27)	1, (2.22)	2, (4.44)
*Enterobacter cloacae*	1, (2.27)	1, (2.22)	1, (2.22)
*Serratia rubidaea*	1, (2.27)	1, (2.22)	1, (2.22)

^a^ Primary stool sample: the stool sample collected during the first 24 h of admission. The primary stool sample was not provided for 1 of the febrile patients (44/45). ^b^ Secondary stool sample: the stool samples were collected after the patients developed a fever. ^c^ Blood sample: blood samples collected from febrile children.

**Table 3:** Antimicrobial resistance patterns of *Enterobacteriaceae* strains isolated from fecal and blood samples in febrile children.

Antibiotics ^a^	Primary Stool Sample ^b^ N = 44 (n, %)	Secondary Stool Samples N = 45 (n, %)	Blood Samples N = 45 (n, %)
IMP (10 µg)	12, (27.27)	19, (42.2)	13, (28.88)
MEM (10 µg)	14, (31.83)	18, (40)	13, (28.88)
AN (30 µg)	13, (29.95)	15, (33)	8, (17.77)
GM (10 µg)	22, (50)	24, (53.3)	11, (24.44)
CAZ (30 µg)	29, (65.9)	39, (86.6)	12, (26.66)
LVX (5 µg)	9, (20.4)	15, (33)	4, (8.88)
CTX (30 µg)	33, (75)	37, (82.2)	13, (28.88)
CEF (30 µg)	23, (52.27)	26, (57.5)	12, (26.66)
AZT (30 µg)	29, (65.9)	32, (71.1)	11, (24.44)
TGC (15 µg)	3, (6.8)	7, (15.5)	3, (6.66)

^a^ IMP (Imipenem), MEM (Meropenem), AN (Amikacin), GM (Gentamycin), CAZ (Ceftazidime), LVX (Levofloxacin), CEF (Cefepime), CTX (Cefotaxime), AZT (Aztreonam), TGC (Tigecycline). ^b^ The primary stool sample was not provided for 1 of the febrile patients (44/45).

**Table 4:** Demographic data of immunocompromised children with fever and the frequency of CR*E* ^a^ in a pediatric hospital in Tehran.

Variables	Frequency (n = 45)	CR*E* (n = 14)	*p*-Value
**Gender**			0.99
Female	20 (20/45, 44.4%)	6 (6/20, 30%)	
Male	25 (25/45, 55.5%)	8 (8/25, 32%)	
**Age**			0.99
1–5 years old	22 (22/45, 48%)	7 (7/22, 31.81%)	
6–10 years old	11 (11/45, 24%)	3 (3/11, 0.27%)	
11–15 years old	12 (12/45, 26%)	4 (4/12, 0.33%)	
**Fever day**			**0.022 ^b^**
7 days (first week)	36 (36/45, 80%)	8 (8/36, 0.22%)	
14 days (second week)	5 (5/45, 11%)	3 (3/5, 0.6%)	
21 days (third week)	4 (4/45, 8%)	3 (3/4, 0.75%)	
**LOS**			**0.018 ^b^**
7 days (first week)	13 (13/45, 28.8%)	2 (2/13, 15.38%)	
14 days (second week)	17 (17/45, 37.7%)	3 (3/17, 17.64%)	
Over 14 days (third week)	15 (15/45, 33.3%)	9 (9/15, 0.6%)	
**Underlying diseases**			0.83
AML	12 (12/45, 26%)	0	
ALL	6 (4/45, 13%)	1 (1/6, 16.66%)	
Burkitt’s lymphoma	3 (3/45, 6.6%)	3 (3/3, 100%)	
Neuroblastoma	5 (5/45, 11.1%)	2 (2/5, 0.4%)	
CGD	2 (2/45, 4.4%)	0	
Histiocytic disorders	1 (1/45, 2.2%)	0	
Lymphoma	7 (7/45, 15.5%)	3 (3/7, 42.85%)	
PNH	1 (1/45, 2.2%)	0	
RMS	1 (1/45, 2.2%)	1 (1/1, 100%)	
Wilms tumor	3 (3/45, 6.6%)	1 (1/3, 33.33%)	
HLH	1 (1/45, 2.2%)	0	
YST	1 (1/45, 2.2%)	1 (1/1, 100%)	
RES	1 (1/45, 2.2%)	1 (1/1, 100%)	
Aplastic anemia	1 (1/45, 2.2%)	1 (1/1, 100%)	
**Ward**			0.46
BMT	11 (11/45, 24.44%)	2 (2/11, 18.18%)	
Oncology	34 (34/45, 75.5%)	12 (12/34, 35.29%)	
**Prophylaxis antibiotics**			0.99
Carbapenem based regime	19 (19/45, 42.22%)	5 (5/19, 26.31%)	
Non-carbapenem based regime	26 (26/45, 57.77%)	9 (9/26, 34.61%)	
**Alternative antibiotic**			0.99
Carbapenem based regime	20 (20/45, 44.44%)	6 (6/20, 3%)	
Non-carbapenem based regime	25 (25/45, 55.55%)	8 (8/25, 32%)	
**Chemotherapy drug**			**0.03 ^b^**
Cyclophosphamide + Adriamycin + vincristine	10 (10/45, 22.25%)	1 (1/10, 10%)	
Cytosar	8 (8/45, 17.7%)	3 (3/8, 37.5%)	
GCSF	7 (7/45, 15.5%)	2 (2/7, 28.57%)	
Cyclophosphamide + vincristine	7 (7/45, 15.5%)	4 (4/7, 57.14%)	
Doxorubicin + cyclophosphamide + vincristine	5 (5/45, 11.1%)	1 (1/5, 20%)	
Fludarabin + cytocar	4 (4/45, 8.8%)	1 (1/4, 25%)	
6MP	4 (4/45, 8.8%)	2 (2/14, 50%)	
**Death**			**0.02 ^b^**
Yes	11 (11/45, 24.4%)	7 (7/11, 63.63%)	
No	34 (34/45, 75.5%)	7 (7/34, 20.58%)	

(^a^) CR*E*: Carbapenem-resistant *Enterobacteriaceae*; LOS; Length of stay, AML; Adult acute myeloid leukemia, ALL; Acute lymphocytic leukemia, CGD; Chronic granulomatous disease, PNH; Paroxysmal nocturnal hemoglobinuria, RMS; Rhabdomyosarcoma, HLH; Hemophagocytic lymphohistiocytosis, YST; Yolk sac tumor, RES; Ewing Sarcoma, Alternative antibiotic; the antibiotic alternated after patients got fever. (^b^) *p* values represent significant differences between fever onset during the first week of hospitalization compared with longer periods; CR*E* bacteremia among children who presented >2 weeks LOS; inverse correlation between chemotherapy regimens and bacteremia; and death rate comparison between patients with and without CR*E* bacteremia. Numbers in bold represent *p*-Values that are statistically significant.

## Data Availability

The data presented in this study are available upon reasonable request from the corresponding authors.

## Supplementary Materials

The following supporting information can be downloaded at: https://www.mdpi.com/article/10.3390/tropicalmed8080402/s1, Figure S1: Diagram of sampling stages from immunocompromised children.
