# Supplementary material for: Endogenous Bacteremia Caused by Intestinal Colonization of Carbapenem-Resistant Enterobacteriaceae (CRE) in Immunocompromised Children
Source: Trop Med Infect Dis. 2023 Aug 7;8(8):402. doi: 10.3390/tropicalmed8080402 (PMC10458169; doi:10.3390/tropicalmed8080402)
Supplement: Supplementary file 1 [file tropicalmed-08-00402-s001.zip › tropicalmed-2486325-SI.pdf]

Tropical Medicine and Infectious Disease

Supplementary Materials of Endogenous Bacteremia Caused by Intestinal Colonization of Carbapenem-Resistant *Enterobacteriaceae* (CRE) in Immunocompromised Children

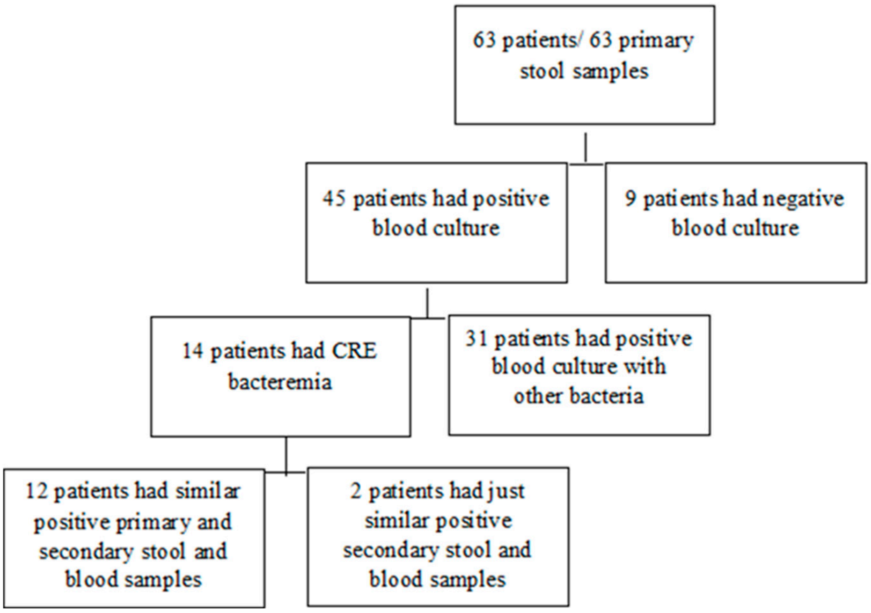

Figure S1. Diagram of sampling stages from immunocompromised children.
